# Grapevine Botryosphaeria dieback fungi have specific aggressiveness factor repertory involved in wood decay and stilbene metabolization

**DOI:** 10.1371/journal.pone.0188766

**Published:** 2017-12-20

**Authors:** Elodie Stempien, Mary-Lorène Goddard, Kim Wilhelm, Céline Tarnus, Christophe Bertsch, Julie Chong

**Affiliations:** 1 Université de Haute-Alsace, Laboratoire Vigne, Biotechnologies et Environnement, Colmar, France; 2 Université de Haute-Alsace, Laboratoire de Chimie Organique et Bioorganique, Mulhouse, France; Universita degli Studi di Pisa, ITALY

## Abstract

Grapevine trunk diseases: Eutypa dieback, esca and Botryosphaeria dieback, which incidence has increased recently, are associated with several symptoms finally leading to the plant death. In the absence of efficient treatments, these diseases are a major problem for the viticulture; however, the factors involved in disease progression are not still fully identified. In order to get a better understanding of Botryosphaeria dieback development in grapevine, we have investigated different factors involved in *Botryosphaeriaceae* fungi aggressiveness. We first evaluated the activity of the wood-degrading enzymes of different isolates of *Neofusicoccum parvum* and *Diplodia seriata*, two major fungi associated with Botryosphaeria dieback. We further examinated the ability of these fungi to metabolize major grapevine phytoalexins: resveratrol and δ-viniferin. Our results demonstrate that *Botryosphaeriaceae* were characterized by differential wood decay enzymatic activities and have the capacity to rapidly degrade stilbenes. *N*. *parvum* is able to degrade parietal polysaccharides, whereas *D*. *seriata* has a better capacity to degrade lignin. Growth of both fungi exhibited a low sensitivity to resveratrol, whereas δ-viniferin has a fungistatic effect, especially on *N*. *parvum* Bourgogne S-116. We further show that *Botryosphaeriaceae* are able to metabolize rapidly resveratrol and δ-viniferin. The best stilbene metabolizing activity was measured for *D*. *seriata*. In conclusion, the different *Botryosphaeriaceae* isolates are characterized by a specific aggressiveness repertory. Wood and phenolic compound decay enzymatic activities could enable *Botryosphaeriaceae* to bypass chemical and physical barriers of the grapevine plant. The specific signature of *Botryosphaeriaceae* aggressiveness factors could explain the importance of fungi complexes in synergistic activity in order to fully colonize the host.

## Introduction

Since 1974, European viticulture is facing a new class of severe diseases called grapevine trunk diseases. The major grapevine trunk diseases, *i*.*e*. esca, Eutypa dieback and Botryosphaeria dieback, caused by a complex of xylem-inhabiting fungi, are very harmful and therefore an important matter of concern for the viticulture. Indeed, grapevine trunk diseases generate severe yield reduction in vineyards (for review, see [1] and [2]), causing colossal losses which have been estimated to exceed 1 billion dollars per year [3]. Trunk diseases, which incidence has increased since currently no effective plant protection strategies are available, are associated with several symptoms, such as sectorial and/or central necrosis in woody tissues, brown stripes or cankers, leaf discoloration and withering of inflorescence and berries resulting in long-term death of the plant [4–6].

Grapevine trunk diseases involve different actors and factors whose understanding is not yet complete. Koch's postulates, designed to identify the link between diseases and causal agents, are difficult to demonstrate. Indeed, the reproduction of all symptoms, especially the foliar symptoms, by artificial inoculation is not always verified. In the present work, we focused on Botryosphaeria dieback, which is associated with a wide range of *Botryosphaeriaceae* species [7]. Typical symptoms found in vineyards are foliar discolorations that vary from red to white cultivars, wood discolorations, grey sectorial necrosis and orange/brown area just beneath the bark, leading to long-term plant death [4–7]. More than 30 different *Botryosphaeriaceae* species have been found in vineyards in several countries based on morphological traits and genetic markers [7–11]. *Neofusicoccum parvum* (teleomorph form: *Botryosphaeria parva*; [12,13] and *Diplodia seriata* De Not. (teleomorph form: *Botryosphaeria obtusa* (Schwein.) [14–16] have been especially associated with Botryosphaeria dieback [10,17]. Several authors demonstrated a great variability in the aggressiveness level between *N*. *parvum* and *Diaporthe species* [18]. In detail, toxicity assays on *Vitis* cells and leaves and study of necrosis development after artificial inoculation of *Vitis* canes, showed that *N*. *parvum* is more aggressive than *D*. *seriata* [7,9,19–23].

Aggressiveness of fungi associated with trunk diseases may be related to different fungal activities. Variation in wood-decay abilities and production of phytotoxic compounds are thought to be main factors contributing to trunk disease fungi unique disease symptoms. Mycelia of trunk disease fungi are detected in the trunk but never from the symptomatic leaves or berries of infected plant [24], then it has been hypothesized that toxins secreted by fungi in the trunk are translocated to the leaves and berries via the xylem sap to induce symptoms [25]. Indeed, several phytotoxic secondary metabolites are produced by the main fungi associated with trunk diseases (for review, see [26]). In the case of B. dieback fungi, dihydroisocoumarins are produced by *D*. *seriata*, whereas *N*. *parvum* is characterized by the synthesis of dihydrotoluquinones, epoxylactones, dihydroisocoumarins and hydroxybenzoic acids [23,27].

Concerning wood decay activity, different authors demonstrated that pathogens associated with esca or Eutypa dieback are able to produce wood degradation enzymes in order to gain nutrients and to progress in the trunk. Different authors discovered that wood decay enzymes such as xylanases, cellulases, β-glucanases, chitinases, proteases, oxydases, glucosidases and starch degrading enzymes are produced by *Eutypa lata*, responsible for Eutypa dieback [28–30]. Moreover, it has been demonstrated that *E*. *lata* targets glucose-rich polymers and that after infection, glucose and xylose of the hemicellulose cell wall fraction were depleted both in susceptible (*Vitis vinifera* cv. Cabernet Sauvignon) and tolerant (*V*. *vinifera* cv. Merlot) grapevine cultivars. *Phaeomoniella chlamydospora* and *Phaeoacremonium minimum comb*. *nov*. are two fungi responsible for the esca syndrome in grapevine [30]. It has been reported that *P*. *minimum* possessed enzyme activities responsible for the degradation of polysaccharides (xylanase, exo- and endo-β-1.4-glucanase and β-glucosidase), whereas no ligninase activity was measured [31]. In contrast, none of these enzyme activities was measured in *P*. *chlamydospora*, which was characterized by a pectinolytic activity enabling to circulate through the vessels without degrading the membrane [32,33]. These studies suggest that these two fungi have different strategies of wood colonization.

Recent analysis of the sequenced genomes of major trunk disease associated fungi, including *N*. *parvum* and *D*. *seriata*, revealed an expansion of gene families related to plant cell wall degradation [34]. Approximately 36.6% of the putative secreted peptides in the genomes of eight trunk pathogens were similar to CAZy proteins, with predicted catalytic and carbohydrate-binding domains that can participate in the disassembly of plant cell walls during pathogen attack. The largest superfamily was represented by glycoside hydrolases that include subfamilies involved in the degradation of cellulose and hemicellulose [34].

One of the grapevine responses to wood decay fungi is the production of secondary metabolites called phytoalexins. Phytoalexins are produced after infection with pathogens or recognition of an elicitor in different plant species and can act as markers of resistance [35]. In grapevine, phytoalexins belong essentially to the class of stilbenes synthesized via the phenylpropanoid pathway. The grapevine stilbenes have an antifungal activity [36,37], inhibiting spore germination, fungal penetration, fungal growth and development [38]. The first synthesized stilbene, resveratrol, will be modified in various compounds in grapevine [38,39]. Resveratrol glycosylation will form piceid, which is a non-toxic stilbene that could represent inactive storage form [40,41]. δ- and ε-viniferin are resveratrol dimers synthesized by an oxidation reaction, they are considered as the most toxic forms of stilbenes [42]. Resveratrol methylation will lead to the synthesis of the potent antifungal pterostilbene by a resveratrol *O*-methyltransferase (ROMT) [42,43]. Together with viniferins, pterostilbene is one of the most toxic stilbene form, 10 fold more toxic than resveratrol [42,43].

In the case of grapevine trunk diseases, accumulation of stilbenes including resveratrol, ε-viniferin and two other resveratrol oligomers, has been shown in the brown-red wood, leaves and berries of esca-diseased grapevines [44–47]. In addition, genes encoding *PAL* (Phenylalanine Ammonia Lyase) and stilbene synthase (*STS*), two phenylpropanoid biosynthesis enzymes, were strongly expressed in asymptomatic leaves before the appearance of the esca apoplectic form [48]. Lambert et al. [49] studied the *in vitro* antifungal activity of different stilbenes on different isolates and species of *Botryosphaeriaceae* and demonstrated that resveratrol, pterostilbene and ε-viniferin inhibited the growth of *Botryosphaeriaceae*, especially *N*. *parvum* and *D*. *seriata*. However, this study also reported that fungi associated with esca, *P*. *minimum*, *P*. *chlamydospora* and *Fomitiporia mediterranea* have the ability to grow in media rich in phenolic compounds [49].

The capacity of trunk disease fungi to produce enzymes degrading antifungal metabolites synthesized by the plant cell could play a significant role in the outcome of the interaction between grapevine and pathogens. Oxidation of phenolic compounds can be achieved by laccases, enzymes occuring widely in fungi [50]. *Botrytis cinerea*, the grey mold agent, produces a laccase-like stilbene oxidase that catalyzes the oxidation of resveratrol in different dimer compounds, in particular δ-viniferin, the most abundant resveratrol dimer [51,52].

Enzymes involved in the oxidation of phenolic compounds are also involved in lignin degradation. Lignin and manganese peroxidases are part of the extracellular oxidative system developed by rot fungi to degrade lignin [53]. The esca associated fungi *P*. *chlamydospora* and *P*. *minimum* are characterized by different *in vitro* enzymatic activities such as tannases, laccases and peroxidases activities, and are able to use tannic acid and resveratrol as a carbon source [31,54]. *Fomitiporia mediterranea*, a fungus associated with the white rot of esca, secretes various lignolytic enzymes, such as laccases, peroxidases and phenol oxidases [25]. Analysis of *N*. *parvum* and *D*. *seriata* genomes also demonstrated a significant expansion of gene families associated with specific oxidative functions, which may contribute to lignin degradation [34]. The synthesis of enzymes involved in the degradation of starch and lignocellulose has been reported in *Botryosphaeria* sp. and could be regulated by veratryl alcohol, a secondary metabolite synthesized by fungi [55].

In order to develop effective control strategies against grapevine trunk diseases, it appears necessary to fully identify the factors involved in disease progression and to understand all aspects of their pathogenicity. In a work studying 56 Botryosphaeriales strains, including *N*. *parvum* and *D*. *seriata*, the authors showed that most strains produced cellulases, laccases, xylanases, pectinases, pectin lyases, amylases, lipases and proteases [56]. Here, the aim of our work was to link B. dieback fungi aggressiveness with a capacity to decay grapevine wood and/or a capacity to detoxify phytoalexins. We focused on different strains of *N*. *parvum* and *D*. *seriata*, characterized by different aggressiveness levels on grapevine.

We first investigated the ability of secreted fungi proteins to degrade cell wall polysaccharides and lignin by enzymatic activities. We have previously shown that *Vitis* cells are able to produce stilbenes after detection of secreted proteins from *Botryosphaeriaceae* [57]. In this work, we also studied the activity of resveratrol and δ-viniferin produced by grapevine on *Botryosphaeriaceae* fungi growth and we further investigated the ability of these fungi to detoxify them. Stilbene detoxification activity could be an important component of the pathogenicity mechanism of trunk disease fungi.

## Material and methods

### Fungi

*D*. *seriata* 98.1 and *N*. *parvum* Bourgogne S-116 were obtained from the collection of the IFV (Institut Français de la Vigne et du Vin, Rodilhan, France). *N*. *parvum* Bt-67 was obtained from a single spore collection of the Instituto Superior de Agronomia (Universidade de Lisboa, Portugal). *D*. *seriata* 98.1 was isolated in 1998 in Perpignan as described in Larignon et al. (2001) [58]. *N*. *parvum* Bourgogne S-116 was isolated in 2009 from Chardonnay plants showing decline in nurseries. *N*. *parvum* Bt-67 was isolated from vineyards in Portugal. Fungi were grown in Petri dishes containing PDA (Potato Dextrose Agar) solid medium at 27°C in the dark. The fungi were subcultured every 10 days.

### Isolation of total extracellular proteins

Fungi solid cultures were grown for 10 days, then three mycelia plugs for each fungus (2.5 x 2.5 cm) were introduced in a 500 mL Erlenmeyer flask containing 250 mL liquid culture medium. We used 4 different liquid culture media: malt medium, malt medium supplemented with *V*. *vinifera* cv. Gewurztraminer sawdust, Erickson and Peterson medium [59], and Ericksson and Peterson medium supplemented with *V*. *vinifera* cv. Gewurztraminer sawdust. Liquid cultures were grown at 220 rpm, in the dark and at 28°C. After twenty-one days, culture media were retrieved and sterilized by successive filtrations at 0.8, 0.45 and 0.2 μm.

Total extracellular proteins were precipitated from 250 mL filtered culture medium with 60% (w/v) ammonium sulfate according to [22]. After 2 hours of shaking at 4°C, extracts were centrifuged 30 min at 10 000 rpm and the protein pellets were resuspended in deionized water and dialyzed in 3.5 kDa cutoff tubbing against deionized water for 20 h at 4°C. Collected proteins were freeze dried and resuspended in 3 mL ultra-pure water. The protein concentration was measured with a BioSpec-nano Micro-volume Spectrophotometer (Shimadzu™, Japan) at 280 nm.

### Enzymatic activity of wood degradation

The total extracellular protein extract from the different fungi (2 mg.mL^-1^ total proteins) was used to measure enzymatic activities involved in wood degradation.

Cellulose and arabic gum were used as substrates to measure the cellulase and hemicellulase activity, respectively. Furthermore, *V*. *vinifera* cv. Gewurztraminer sawdust was used as a substrate to determine the total polysaccharide degrading activity. Cellulase and hemicellulase activities result in reducing sugar liberation that was further measured with the colorimetric method using 3.5-dinitrosalicylic acid (DNS) [60]. This colorimetric assay estimates the concentration of reducing sugars containing free carbonyl group (eg. glucose, lactose). When an alkaline solution of 3.5-dinitrosalicylic acid reacts with reducing sugars or other reducing substances, it is converted into 3-amino-5-nitrosalicylic acid with orange-red color.

A calibration curve was performed using glucose solution at different concentrations: 0, 2, 4, 6, 8, 10, 15, 20 mM. For each concentration, 60 μL of glucose solutions were added to 60 μL of DNS (10 g.L^-1^ DNS, 300 g.L^-1^ sodium potassium tartrate, dissolved in sodium hydroxide at 0.4 N) in a 96-well PCR microplate (ThermoFischer Scientific®, USA). The plate was sealed with an aluminium film (131 x 81.3 mm) and incubated for 5 min at 95° C in order to reduce the DNS. Then, 80 μL of each well was deposited in a new 96-well plate (flat bottom, Sterilin, ThermoFischer Scientific®, USA). The absorbance was measured at 540 nm in a microplate spectrophotometric reader (MCC / 340 2.32, Multiskan®). The calibration curve A_540_ = f ([glucose mM]) was plotted on Microsoft Office Excel (Microsfot, USA®). The equation of the curve is y = 0.129x and the linear regression coefficient is R^2^ = 0.9961.

For the measure of enzymatic activity, the protein extracts were first incubated with the different substrates at the same concentration (cellulose, arabic gum and *V*. *vinifera* cv. Gewurztraminer sawdust at 55.55 mg.mL^-1^): 20 μL of each protein extract (containing 40 μg of total proteins) was deposited in a 96-well PCR microplate with a raised skirt (Kisker Biotech GmbH and Co®, Germany) and 180 μL of a buffer solution (50 mM sodium acetate buffer pH 5, 30 μg.mL^-1^ cycloheximide, 40 μg.mL^-1^ tetracycline) containing the different substrates (10 mg per well) were added. Two negative controls containing either only the substrate or the protein extracts were performed. The plate was sealed with an aluminium film (131 x 81.3 mm) and incubated for 3 h at 50°C in a thermocycler (Thermocycler Multither, Benchmark Scientific®, USA) with a shaking of 600 rpm. Then, 140 μL of each mixture were transferred to a 96-well filter plate (1 μm, fiberglass, AcroPrep Advance 96 Fliter plate, PALL®, USA), coupled to a 96-well recovery plate (flat bottom, Greiner Bio -one®, Germany) and centrifuged for 5 min at 800 rpm. Recovery plate contents were transferred to a new 96-well PCR microplate (4titude®Ltd, UK) and 60 μL of DNS were added before sealing the microplate with an aluminium film (131 x 81.3 mm). The plate was incubated for 5 min at 95°C. After incubation, 80 μL of each mixture was deposited in a reading plate and the absorbance at 540 nm was determined using a microplate spectrophotometric reader (MCC / 340 2.32, Multiskan®). The concentration of reducing sugars was calculated from the calibration curve equation, and the concentration of reducing sugars of the negative controls was subtracted to the sample values. The results are expressed as the amount of reducing sugars produced (in mol) per g of proteins.

Laccase activity was determined with the ABTS (2,2′-azino-bis(3-ethylbenzothiazoline-6-sulfonic acid) substrate. ABTS is oxidized by laccase in its corresponding radical cation ABTS^.+^, which produced a blue color. For each test, 100 μg of secreted proteins (2 mg.mL^-1^) were mixed with 50 μL of a solution containing 25 μL ABTS (2 mM) and 25 μL of sodium acetate (100 mM, pH 4.5), and incubated for 30 min at 25°C in a 96-well microplate (flat bottom, Sterilin, ThermoFischer Scientific®, USA). After 30 min, the absorbance at 420 nm was measured with a microplate reader (MCC / 340 2.32, Multiskan®). The obtained absorbance values were converted into enzymatic activity via the ABTS^.+^ extinction coefficient (36,000 M^-1^ cm ^-1^) according to [61].

Manganese peroxidase activity was based on the coupling of MBTH and DMAB in the presence of H_2_O_2_ and Mn^2+^ according to [61]. The reaction in the presence of Mn^2+^ allows to specifically measure the Mn peroxidase activity. We used two solutions, A and B. The solution A contained 0.5 ml of DMAB solution (50 mM), 0.5 mL of MBTH solution (1 mM), 1 mL MnSO_4_–4H_2_O solution (1 mM) and 5 mL of sodium lactate and sodium succinate buffer (100 mM each, pH 4.5) for a final volume of 7 mL. The solution B was identical to the solution A except for the MnSO_4_–4H_2_O which was replaced by a solution of EDTA (2mM). For the manganese peroxidase assay, 140 μL of solution A or B are mixed with 10 μL of H_2_O_2_ (1 mM) and 50 μL of secreted proteins (2mg.mL^-1^) and incubated for 1 hour at 26°C in a 96-well microplate (flat bottom, Sterilin, ThermoFischer Scientific®, USA). Absorbance at 590 nm was measured with a microplate reader (MCC / 340 2.32, Multiskan®). Mn peroxidase activity was obtained by subtracting the absorbance values obtained with solution B to those obtained with solution A, containing MnSO_4_. Absorbances were converted into enzymatic activity via the extinction coefficient (32,000 M^-1^ cm ^-1^).

### Chemicals

*Trans*-resveratrol, lyophilized horseradish peroxidase HRP and hydrogen peroxide (30%) were purchased from Sigma-Aldrich (St Quentin Fallavier, France). *Trans*-δ-viniferin was synthetized according to the procedure described below. *Cis*-isomers of resveratrol and δ-viniferin were obtained by photoisomerization at 350 nm using a Rayonet photochemical reactor (Southern New England Ultraviolet Co.). 25 mM stock solutions of *trans*-stilbenes were prepared in anhydrous DMSO (Dimethyl Sulfoxide) purchased from Alfa Aesar (Karlsruhe, Germany). LC-MS grade water, methanol and formic acid were purchased from Fisher Scientific (Illkirch, France).

For δ-viniferin synthesis, we used general experimental procedures: Thin layer chromatography (TLC) was performed on Merck 60 F254 silica gel. Silica gel 73–230 mesh (Merck, Darmstadt, Germany) was used for column chromatography. High-resolution electrospray ionization mass spectrometry (HRESI MS) was performed on an Agilent 3100 QTof. FTIR spectra were recorded by ATR technique using Brüker Vertex 70. NMR spectra were recorded at 300 K in deuterated acetone on Brüker Avance 400 MHz spectrometer. Chemical shifts (δ) are indicated in ppm and coupling constants (J) in Hz.

δ-viniferin was synthesized according to a procedure inspired by [62]. 0.7 mg of HRP in 700 μL of phosphate buffer (20 mM pH8) were added to a solution of *trans*-resveratrol (1.76 mmol, 400 mg) in 1:1 v/v acetone/phosphate buffer 20 mM pH 8 (10 mL.mmol^-1^). The mixture was stirred at 40°C and 360 μL of 30% H_2_O_2_ were added during 40 min. Then, the reaction mixture was extracted with EtOAc. The organic layer was washed with water, dried over anhydrous magnesium sulphate and concentrated. The crude product was purified on silica gel column and eluted with dichloromethane:acetone 75:25 (1% v/v NET_3_). δ-viniferin was obtained as a yellow amorphous powder (60.7 mg, 46% yield). HRESI MS (-) *m/z* 453.1320 [M-H]^-^ (calcd for C_28_H_21_O_6_ 453.1344) 499.1389 [M+HCOOH-H]^-^ calcd for C_29_H_22_O_8_ 498.1315); FTIR (ATR) v_max_ cm−1: 3308, 1596, 1487, 1341, 1234, 1149; ^1^H NMR (400 MHz, acetone-d6) δ = 8.55 (1 H, br. s), 8.29 (1 H, s), 8.26 (1 H, s), 7.43 (1 H, dd, *J* = 1.8, 8.4 Hz), 7.24 (3 H, m), 7.06 (1 H, d, *J* = 16.4 Hz), 6.88 (4 H, m), 6.53 (2 H, d *J* = 2.0 Hz), 6.28 (1 H, t, *J* = 2.3 Hz), 6.25 (1 H, t, *J* = 2.0 Hz), 6.198 (2 H, d, *J* = 2.3 Hz), 5.45 (1 H, d, *J* = 8.0 Hz), 4.46 (1 H, d, *J* = 8.0 Hz). ^13^C NMR (100 MHz, acetone-d6) δ = 160.7, 159.9, 159.7, 158.6, 145.3, 140.8, 132.6, 132.3, 131.8, 129.2, 128.7, 128.7, 127.3, 124.0, 116.3, 110.2, 107.5, 105.8, 102.8, 102.5, 94.1, 57.9. Spectral data are in agreement with the literature [63].

### Fungi growth inhibition tests

The fungistatic activity of resveratrol and δ-viniferin was tested on *N*. *parvum* Bourgogne S-116, *N*. *parvum* Bt-67 and *D*. *seriata* 98.1. Stilbenes were dissolved in anhydrous DMSO. Stilbenes (50 μM or 250 μM final concentration) or the same volume of DMSO (negative control) was added to PDA medium at a final concentration of 1%. After 10 days of growth on PDA alone medium, mycelia plugs of 5 mm diameter were transferred at the center of a new Petri Dish containing PDA medium and stilbenes in DMSO or DMSO alone. Petri dishes were incubated at 26°C in the dark and pictures were taken each day from 48 h after inoculation until Petri dish saturation. Mycelia area was measured with ImageJ software [64]. For each condition, 3 technical replicates and 2 biological replicates were performed.

### Metabolite extraction

PDA culture medium of fungi grown in the presence or absence of stilbenes (concentration of 50 μM) was extracted at 3, 6, 10 or 11 days with ethyl acetate (3 x 50 mL, 24h with stirring at room temperature). The supernatant was filtered and evaporated with a rotary evaporator. The extract was dissolved in 1 mL methanol, filtered with a 0.2 μm PTFE filter, and kept at -20°C until LC-MS analysis. A 10-fold dilution in methanol was used for analysis.

### LC-MS analysis

The analytical system used was High Performance Liquid Chromatography Agilent 1100 series coupled to Agilent 6510 accurate-mass Quadrupole-Time of Flight (Q-TOF) Mass spectrometer with ESI interface in negative ionization mode (Agilent Technologies, California, USA). Data acquisition system software was Agilent MassHunter version B.02.00. A Zorbax SB-C18 column (3.1×150 mm, ∅ 3.5 μm), equipped with a 2.1x12.5 mm ∅ 5 μm Zorbax Eclipse plus C18 precolumn (Agilent Technologies), was used at 35°C and the injected volume was 2 μL. The elution gradient was carried out with binary solvent system consisting of 0.1% formic acid in H_2_O (solvent A) and 0.1% formic acid in MeOH (solvent B) at a constant flow-rate of 0.35 mL.min^-1^. The gradient elution program was as follows: 0−3.0 min, 5% B; 3.0−23.0 min, up to 100% B; held for 10.0 min, followed by 7 min of stabilization at 5% B.

The mass spectrometer operated by detection in scan mode with the following settings: drying gas 13.0 L.min^-1^ at 325°C; nebulizer pressure 35 psi; capillary voltage -3500 V, fragmentor 150 V. Negative mass calibration was performed with standard mix G1969-85000 (Agilent Technologies).

Data processing was performed with Agilent MassHunter Qualitative and Quantitative software version B.07.00. Absolute stilbene contents were estimated from external calibration curves prepared with pure standards.

### Statistical analysis

Data were analyzed by using a multifactorial ANOVA and a multiple comparison of means using the Tukey test (p ≤ 0,05) performed with R 3.3.2 software (R Development Core Team 2017; [65]).

## Results

### Polysaccharide degrading enzymatic activities of *N*. *parvum* and *D*. *seriata*

**Fig 1:**
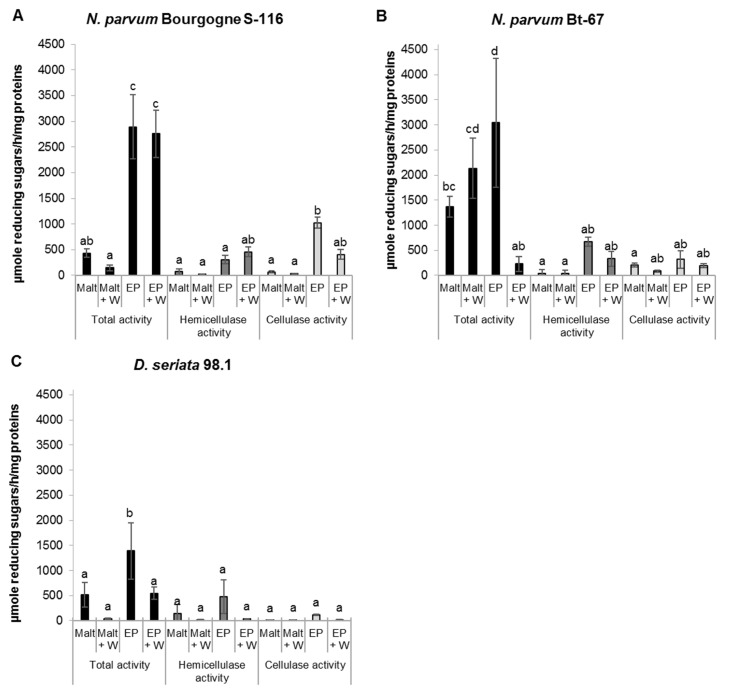
Polysaccharide degrading enzymatic activities. Glucose liberation resulting from cellulase, hemicellulase and total activities of secreted proteins from *N*. *parvum* Bourgogne S-116 (A), *N*. *parvum* Bt-67 (B) and *D*. *seriata* 98.1 (C), was measured with a colorimetric method using dinitrosalicylic acid (DNS). Total proteins were isolated from culture filtrates of fungi grown in liquid malt medium (Malt), malt medium supplemented with *V*. *vinifera* sawdust (Malt + W), Erickson and Peterson medium (EP) and Erickson and Peterson medium supplemented with *V*. *vinifera* sawdust (EP + W). Values are means and SD of three biological replicates, each calculated from the mean of three technical replicates. Means with a same letter are not significantly different at *p*≤0.05 (Tukey Contrasts).

We first evaluated three different enzymatic activities: cellulase, hemicellulase, and total polysaccharide degrading activities of secreted proteins produced by *N*. *parvum* Bourgogne S-116, *N*. *parvum* Bt-67 and *D*. *seriata* 98.1 (Fig 1). We observed a higher total polysaccharide degrading activity with grapevine sawdust as substrate compared to the activity on other substrates (arabic gum and cellulose). Total polysaccharide degrading enzymatic activity was also higher for *N*. *parvum* compared to *D*. *seriata* (Fig 1) and higher when fungi were cultured in EP medium (minimum medium) compared to malt (rich) medium. Proteins secreted by *N*. *parvum* Bourgogne S-116 presented weak enzymatic activities when the fungus was grown in malt medium, whereas total and cellulase activities were significantly enhanced in minimum EP medium (Fig 1A). In contrast, proteins secreted by *N*. *parvum* Bt-67 presented similar enzymatic activities when the fungus was grown in rich malt medium or minimum EP medium (Fig 1B). Finally, *D*. *seriata* 98.1 secreted proteins had lower enzymatic activities (total, cellulase, hemicellulase) (Fig 1C). Generally, the enrichment of fungi culture medium with *V*. *vinifera* sawdust does not have a significant effect on the protein activities, except for *N*. *parvum* Bourgogne S-116 (Fig 1A).

### Lignin degrading enzymatic activities of *N*. *parvum* and *D*. *seriata*

**Fig 2:**
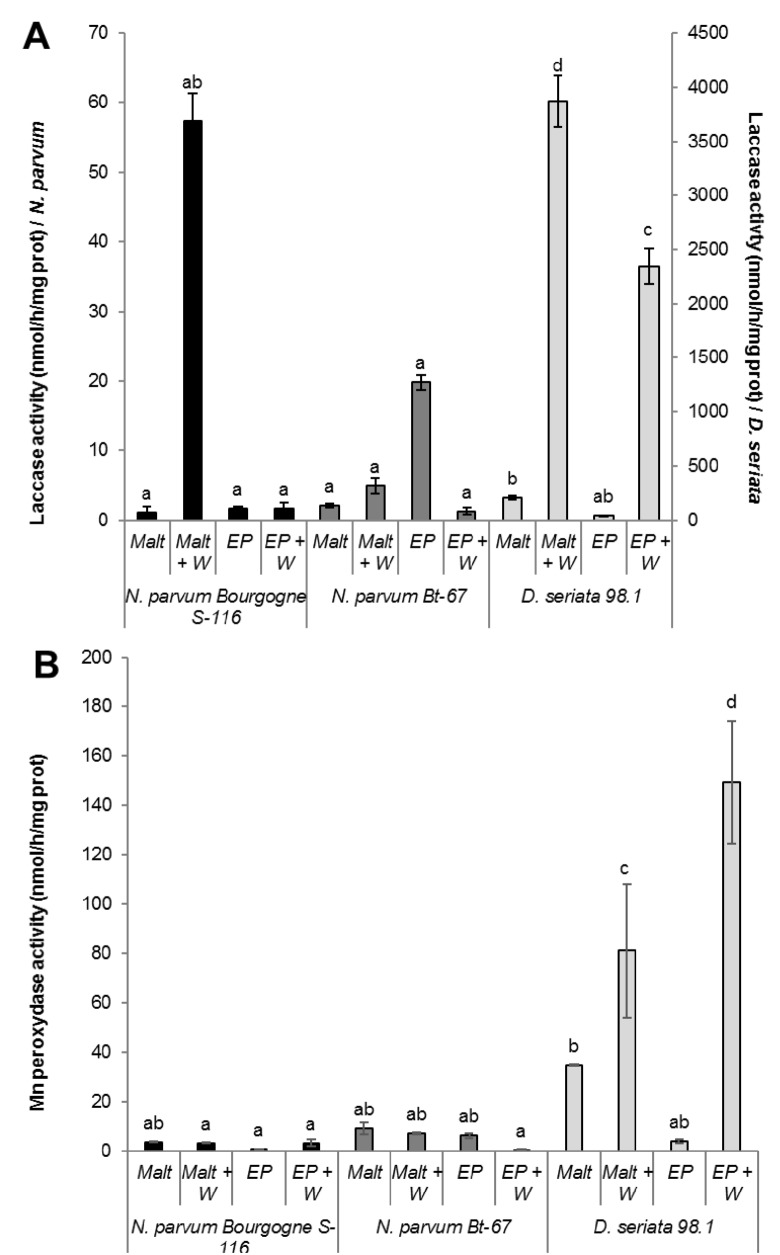
**(A) Laccase activity.** Oxidation of ABTS in the radical cation ABTS^.+^ was measured with total secreted proteins from *N*. *parvum* Bourgogne S-116, *N*. *parvum* Bt-67 and *D*. *seriata* 98.1. Total proteins were isolated from culture filtrates of fungi grown in malt medium (Malt), malt medium supplemented with *V*. *vinifera* sawdust (Malt + W), Erickson and Peterson medium (EP) and Erickson and Peterson medium supplemented with *V*. *vinifera* sawdust (EP + W). **(B) Manganese peroxidase activity.** Oxidation coupling of MBTH/DMAB was measured in presence of H_2_O_2_ and Mn^2+^. Total proteins were isolated from culture filtrates of fungi grown in liquid malt medium (Malt), malt medium supplemented with *V*. *vinifera* sawdust (Malt + W), Erickson and Peterson medium (EP) and Erickson and Peterson medium supplemented with *V*. *vinifera* sawdust (EP + W). Values are means and SD of three biological replicates, each calculated from the mean of two technical replicates. Means with a same letter are not significantly different at *p*≤0,05 (Tukey Contrasts).

It is assumed that lignin degradation is initiated by the attack of laccases, manganese peroxidases and lignin peroxidases. Interestingly, laccase activity was significantly stronger for *D*. *seriata* 98.1 secreted proteins, especially when cultured in malt or EP media supplemented with *V*. *vinifera* sawdust (Fig 2A). In *N*. *parvum* Bourgogne S-116, the laccase activity was also enhanced when fungus was grown in malt medium supplemented with sawdust. The lowest laccase activity was measured for *N*. *parvum* Bt-67 (Fig 2A). Overall, the laccase activity measured in *D*. *seriata* was approximately 100-fold higher compared to *N*. *parvum*. We also evaluated manganese peroxidase activity of *Botryosphaeriaceae* secreted proteins with the MBTH/DMAB colorimetric method. As seen for laccase activity, manganese peroxidase activity was significantly higher for *D*. *seriata* 98.1 secreted proteins and especially when grown in malt or EP media supplemented with *V*. *vinifera* sawdust (Fig 2B). The manganese peroxidase activity measured in *D*. *seriata* was approximately 15-fold higher compared to *N*. *parvum*. In contrast with laccase activity, the lowest Mn peroxidase activity was measured for *N*. *parvum* Bourgogne S-116 and not for N. *parvum* Bt-67.

### δ-viniferin has fungistatic activity on *N*. *parvum*

**Fig 3:**
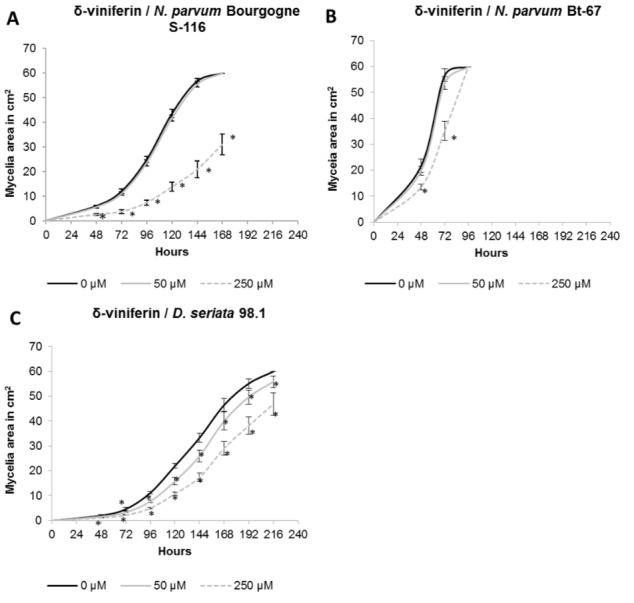
Effect of δ-viniferin on *Botryosphaeriaceae*. The δ-viniferin was tested at a final concentration of 50 μM (grey lines) and 250 μM (gray dotted lines) on *N*. *parvum* Bourgogne S-116 (A), *N*. *parvum* Bt-67 (B) and *D*. *seriata* 98.1 (C) growth. δ-viniferin was dissolved in DMSO and negative control was performed by adding DMSO alone. Values are means and SD of two biological replicates, each calculated from the mean of three technical replicates. Means with a * are significantly different from control at *p* ≤ 0.05 (Tukey Contrasts).

We have previously shown that δ-viniferin is the most abundant stilbene produced after treatment of grapevine suspension cells with *Botryosphaeriaceae* secreted proteins. To test if this compound could be active in inhibiting *Botryosphaeriaceae* mycelial growth, δ-viniferin was added to the solid culture medium of *N*. *parvum* Bourgogne S-116, *N*. *parvum* Bt-67 and *D*. *seriata*, at 50 and 250 μM. Activity of δ-viniferin was compared to the activity of resveratrol which was added at the same concentrations. The addition of 250 μM δ-viniferin in the fungi culture medium resulted in a significant inhibition of *N*. *parvum* Bourgogne S-116 mycelial growth from 48 to 168 hours after inoculation (50% inhibition at 168 h compared to control; Fig 3A). δ-viniferin also induced a weaker but significant inhibition of *D*. *seriata* 98.1 growth both at 50 μM (7% inhibition at 216 h compared to control, Fig 3C) and 250 μM (22% inhibition at 216 h compared to control, Fig 3C). δ-viniferin only induced a slight delay in *N*. *parvum* Bt-67 mycelial growth (30% inhibition at 72 h and 250 μM compared to control, Fig 3B) and after 96 h, this inhibition was no more observable (Fig 3B).

**Fig 4:**
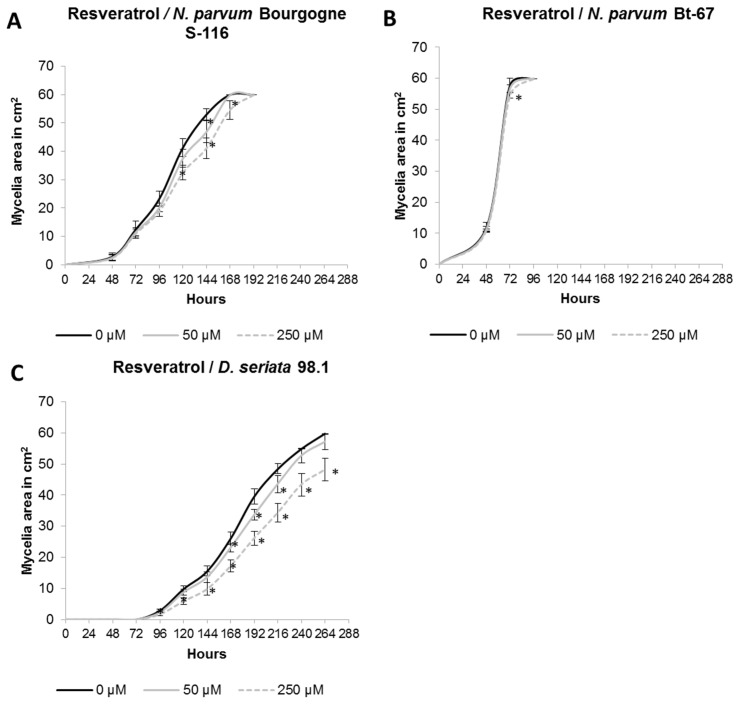
Effect of resveratrol on *Botryosphaeriaceae*. The resveratrol was tested at a final concentration of 50 μM (grey lines) and 250 μM (gray dotted lines) on mycelial growth of *N*. *parvum* Bourgogne S-116 (A), *N*. *parvum* Bt-67 (B) and *D*. *seriata* 98.1 (C). Negative control was performed by adding DMSO instead of resveratrol. Values are means and SD of two biological replicates, each calculated from the mean of three technical replicates. Means with a * are significantly different from control at *p* ≤ 0,05 (Tukey Contrasts).

In contrast to δ-viniferin, resveratrol only induced a delay in the mycelial growth for both *N*. *parvum* isolates, and at the end of the experiment, no inhibition was observable (Fig 4A and 4B). However, for *D*. *seriata* 98.1, we observed a slight but significant inhibition after the addition of resveratrol at a final concentration of 250 μM (19.5% inhibition compared to control at 264 h; Fig 4C).

**Fig 5:**
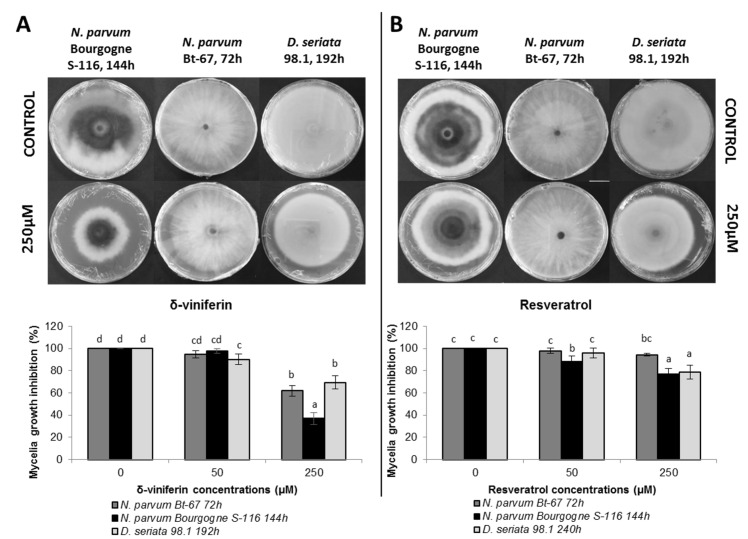
Comparison of the effect of δ-viniferin and resveratrol on *Botryosphaeriaceae*. Effect of δ-viniferin (A) and resveratrol (B) on mycelial growth of *N*. *parvum* BourgogneS-116, *N*. *parvum* Bt-67 and *D*. *seriata* 98.1. Pictures were taken at different time points, when the fungi have nearly colonized all the Petri dish for control conditions (one day before saturation). Values are means and SD of two biological replicates, each calculated from the mean of three technical replicates. Means with a same letter are not significantly different at *p*≤0.05 (Tukey Contrasts).

To take into account the difference of growth rate between the different fungi strains in the comparison of δ-viniferin and resveratrol activities, mycelium surface was measured when the fungi have nearly colonized all the petri dish for control conditions (one day before saturation). The results are similar to those obtained in Figs 3 and 4. It appears that δ-viniferin has a strong effect in inhibiting *N*. *parvum* Bourgogne S-116 growth, a weak effect on *D*. *seriata* and no significant effect on *N*. *parvum* Bt-67 (Fig 5A). Concerning resveratrol, it shows a low activity on both *N*. *parvum* Bourgogne S-116 and *D*. *seriata* 98.1 growth, whereas *N*. *parvum* Bt-67 is not significantly affected (Fig 5B). In conclusion, *N*. *parvum* Bourgogne S-116 is the most susceptible to stilbene activity, followed by *D*. *seriata* and *N*. *parvum* Bt-67.

### *Botryosphaeriaceae* are able to metabolize resveratrol and δ-viniferin

To know if *Botryosphaeriaceae* fungi are able to metabolize stilbenes, we further analyzed the evolution of resveratrol and δ-viniferin (initially added at 50 μM) remaining in the fungi culture medium by LC-MS. Since degradation also occurred in the control culture medium (without fungi), quantities of remaining stilbenes were calculated as a percentage of the quantity found in control culture medium after 3, 6 and 11 days of culture.

**Fig 6:**
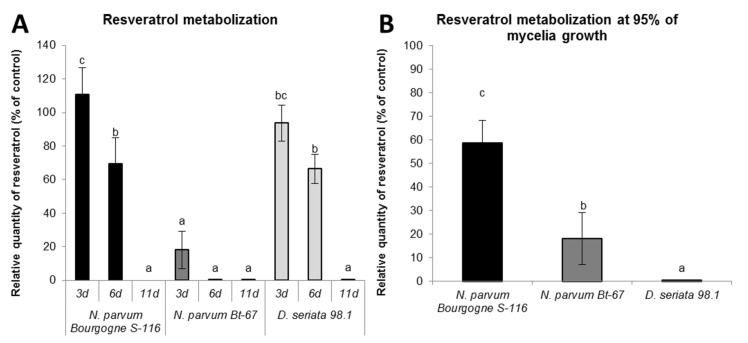
Resveratrol metabolization by *Botryosphaeriaceae* fungi. Resveratrol (initially added at 50 μM) was quantified by LC-MS in PDA medium extract of *N*. *parvum* Bourgogne S-116, *N*. *parvum* Bt-67 and *D*. *seriata* 98.1. The resveratrol quantity is expressed in relative quantity (% of control) compared to control medium without fungi at different time points (3, 6 and 11 days; A), or when fungi have saturated 95% of the Petri Dish surface (B). Values are means and SD of three biological replicates, each calculated from the mean of three technical replicates. Means with a same letter are not significantly different at *p*≤0.05 (Tukey Contrasts).

The Fig 6A shows a rapid decrease in resveratrol for all fungi, that has the same pattern for *N*. *parvum* Bourgogne S-116 and *D*. *seriata* 98.1: approximately 30% of resveratrol found in control culture medium was degraded after 6 days and after 11 days a very low quantity of resveratrol was detected. In *N*. *parvum* Bt-67 medium, there is already a low level of resveratrol after 3 days of growth. Since the growth rate is different between the different *Botryosphaeriaceae*, the Fig 6B shows the relative quantity of resveratrol remaining when the fungus is at 95% of the petri dish saturation. We observed significant differences in the remaining resveratrol between the fungi (*p* ≤ 0,0001). In the medium of *D*. *seriata* 98.1, there is almost no more remaining resveratrol (0.45% of initially added resveratrol), in contrast to *N*. *parvum* Bt-67 medium where 18% of added resveratrol was detected. In *N*. *parvum* Bourgogne S-116 medium, remaining resveratrol quantity is much higher compared to *D*. *seriata* (59%,Fig 6B).

**Fig 7:**
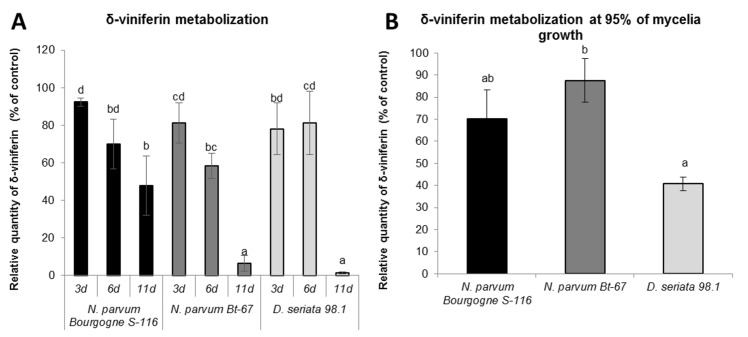
δ-viniferin metabolization by *Botryosphaeriaceae* fungi. δ-viniferin (initially added at 50 μM) was quantified by LC-MS in PDA medium extract of *N*. *parvum* Bourgogne S-116, *N*. *parvum* Bt-67 and *D*. *seriata* 98.1. The δ-viniferin quantity is expressed in relative quantity (% of control) compared to control medium without fungi at different time points (3, 6 and 11 days: A), or when fungi have saturated 95% of the Petri Dish surface (B). Values are means and SD of three biological replicates, each calculated from the mean of three technical replicates. Means with a same latter are not significantly different at *p*≤0,05 (Tukey Contrasts).

Concerning δ-viniferin, Fig 7A shows a decrease over time in the medium of the two *N*. *parvum* isolates and of *D*. *seriata* 98.1. However, δ-viniferin metabolization seems less rapid compared to resveratrol. The reduction in δ-viniferin has the same pattern for the two *N*. *parvum* isolates at 3 days (approximately 85% remaining) and 6 days (65% remaining). After 11 days, a significant proportion of δ-viniferin was measured for *N*. *parvum* Bourgogne S-116 (48% of control), whereas almost all δ-viniferin was metabolized for *N*. *parvum* Bt-67 (6.4% of control). For *D*. *seriata* 98.1, the quantity of δ-viniferin was stable from 3 to 6 days of growth (80% of control) and drop after 6 days (1.5% of control). Measures of remaining δ-viniferin at nearly Petri dish saturation (Fig 7B) demonstrated a less marked difference between the fungi (*p* ≤ 0.05). However, the medium of *D*. *seriata* 98.1 contained the lowest quantity of remaining δ-viniferin compared to *N*. *parvum* isolates.

## Discussion

In this work, we first investigated the wood degradation capacity of *N*. *parvum* and *D*. *seriata*, two major fungi associated with Botryosphaeria dieback, focusing on their extracellular enzymatic material. We also investigated the effect of two major grapevine phytoalexins, resveratrol and δ-viniferin on the growth of these trunk disease fungi. These phytoalexins have been previously described as inefficient against some Botryosphaeria isolates [66,49]. The ability of *Botryosphaeriaceae* fungi to metabolize grapevine stilbenes was thus further studied.

In forest ecosystems, fungi acting as decomposers play major roles in dead wood degradation mediated by extracellular enzymatic systems. Decomposer fungi produce a wide range of extracellular lignolytic enzymes and are divided in three major groups according to their mode of attack on the woody cell walls: soft-rot fungi, brown-rot fungi and white-rot fungi (for review, see [59]). It has been reported that soft rot fungi primarily degrade the carbohydrate components of cell walls and lignin to a lesser extent [67]. In the case of trunk diseases, *E lata* is the only fungus that was classified as a soft rot, a term used to describe all forms of decay caused by ascomycetes [30].

There are few reports on the characterization of enzymatic wood-degrading activity of B. dieback pathogens, although it could bring to light some elements regarding the aggressiveness of *N*. *parvum* and *D*. *seriata*. In a previous study, different authors studied the enzymatic arsenal of 56 strains of Botryosphaeriales, including *N*. *parvum* and *D*. *seriata* [56]. They showed that most strains produced cellulases, laccases, xylanases, pectinases, pectin lyases, amylases, lipases and proteases. However, these enzymatic activities were not associated with any particular species or phylogenetic group [56].

In the present study, we compared secreted wood degradation enzymatic activities of different isolates of B. dieback fungi, with different aggressiveness levels. According to [56], we show that both *N*. *parvum* and *D*. *seriata* are able to degrade the carbohydrate components of cell walls and exhibit hemicellulase and cellulase activities, that could explain at least 60% of the wood decay (cellulose and hemicellulose) [68]. Our data also show that *N*. *parvum* has higher total enzymatic activity of polysaccharide degradation compared to *D*. *seriata*. Compared to *D*. *seriata*, total polysaccharide degrading activity was higher in *N*. *parvum* Bourgogne S-116 grown in EP and EP supplemented with wood medium and in *N*. *parvum* Bt-67 grown in malt, malt supplemented with wood and EP media (Fig 1). This result could be related to a higher growth of *N*. *parvum* after artificial wood inoculation already shown by various authors [19,20,69,9,70,71,23,72]. Our results are also consistent with the trunk disease fungi genome analysis of Morales et al [34], showing that ascomycete trunk pathogens have a wider array of enzymes that target cellulose and hemicellulose compared to other grapevine pathogens. In this study, proteins secreted by both *N*. *parvum* and *D*. *seriata* exhibited a higher enzymatic activity when grown in a minimum medium (EP), in comparison to a rich medium (malt), supposing that adaptation to a poor environment goes through a strongest enzymatic activity, in order to exploit and extract efficiently the nutrients. This was especially significant for *N*. *parvum* Bourgogne S-116, as the total activity was 5 to 8 fold higher in EP medium compared to activity in malt medium.

Concerning lignin degradation, it is assumed that it is initiated by the attack of lignin peroxidase, manganese peroxidase and laccase secreted by white rot fungi [61]. In our study, we were also interested in laccase and Mn peroxidase activities of *Botryosphaeriaceae* secreted proteins. Strikingly, a significantly stronger laccase activity was measured for *D*. *seriata* compared to *N*. *parvum*. Compared to *N*. *parvum*, laccase activity was approximately 100 fold higher for *D*. *seriata* grown in malt or EP medium supplemented with wood. Concerning *N*. *parvum*, laccase activity was higher in Bourgogne S-116 isolate compared to Bt-67 (Fig 2A). Whereas addition of grapevine sawdust to the fungi culture medium did not induce a higher carbohydrate degrading enzymatic activity, it significantly enhanced both laccase and Mn peroxidase activities, especially for *D*. *seriata*. In another study assessing different extracellular enzymatic activities involved in the wood decaying process in different rot type fungi, addition of sawdust did not result in higher enzymatic activities [61].

Characterization of laccase activity in *Botryosphaeriaceae* is consistent with genome analysis of *N*. *parvum* and *D*. *seriata* that revealed an expansion of *auxiliary activity CAZymes 1* genes, encoding multicopper oxidases including laccases, which participate in the deconstruction of lignocellulosic material [34]. A previous study also showed that some *Botryosphaeria* sp. are able to degrade lignocellulose. Moreover lignin degradation could be enhanced by veratryl alcohol, a fungi metabolite, via laccase regulation [55].

Our study also underlines that even if a similar gene expansion was evidenced in both *N*. *parvum* and *D*. *seriata*, a higher laccase activity was measured in *D*. *seriata*. We also show that *D*. *seriata* is characterized by a stronger Mn peroxidase activity, approximately 10 fold higher compared to *N*. *parvum* (Fig 2B). Laccases and peroxidases could be involved in degradation of new lignin and polyphenolic compounds deposited in response to infection. Phenol oxidases produced by *D*. *seriata* could thus participate in breakdown of newly wall bound lignin after infection. It is interesting to point out that association of two major B. dieback fungi (*N*. *parvum* and *D*. *seriata*) could be very efficient in the degradation of the lignocellulosic material.

The enzymatic wood degradation activity has been described for other fungi associated with esca (*P*. *minimum* and *P*. *chlamydospora*) and for *E*. *lata*, responsible for Eutypa dieback. *E*. *lata* was shown to target carbohydrate polymers of cell walls [30]. Concerning esca disease, it has been reported that *P*. *minimum* possessed enzyme activities responsible for the degradation of polysaccharides (xylanase, exo- and endo-β-1.4-glucanase and β-glucosidase), whereas no ligninase activity was measured [31]. *P*. *chlamydospora* was characterized by a pectinolytic activity enabling to circulate through the vessels without degrading the membrane [32,33]. Moreover, *F*. *mediterranea*, which is associated with esca white rot symptoms of mature vines, secreted lignolytic enzymes to complete the wood degradation initiated by *P*. *minimum* and *P*. *chlamydospora* [25,73]. For example, a 60 kDa extracellular laccase has been purified from *F*. *mediterranea* and exhibited oxidase activity towards several polyphenolic compounds [74].

In another part of this work, we studied the effect of stilbenes on *Botryosphaeriaceae* fungi growth. It has been previously demonstrated that grapevine stilbenes have an antifungal activity [36,37], inhibiting spore germination, fungal penetration, fungal growth and development [38]. Based on growth inhibition tests *in vitro*, δ-viniferin is one of the most toxic stilbene on grapevine pathogens [38,42]. In a previous study, we have shown that treatment of grapevine cell suspensions with secreted *Botryosphaeriaceae* proteins resulted in a high accumulation of δ-viniferin [52]. In this work, we tried to determine if stilbene production by grapevine represents a real chemical barrier for the progression of B. dieback fungi. Here we show that growth of fungi in the presence of resveratrol (250 μM) resulted in a slight delay for *N*. *parvum* growth and in a weak inhibition for *D*. *seriata* growth (Fig 4). In contrast, addition of 250 μM δ-viniferin in the culture medium significantly inhibited the growth of *N*. *parvum* Bourgogne S-116, (50% inhibition at day 7) and to a lesser extent that of *D*. *seriata* (22% inhibition at day 9). δ-viniferin had no significant activity on *N*. *parvum* Bt-67 strain. These results show that *N*. *parvum* Bourgogne S-116, which was characterized as a very aggressive strain by secreting active toxins triggering cell death in grapevine calli [22], is also the more susceptible to stilbenes, especially δ-viniferin. The ability of *Botryosphaeriaceae* fungi to grow in the presence of stilbenes strongly suggests that these fungi have the ability to metabolize and degrade these metabolites. Indeed, we further demonstrate that all fungi strains were able to degrade almost all the resveratrol added at 50 μM to the culture medium after 11 days (Fig 6). At earlier time point (6 days), approximately 30% of resveratrol was degraded by *N*. *parvum* Bourgogne S-116 and *D*. *seriata* 98.1. Among the three fungi strains, *N*. *parvum* Bt-67 is the fungi that metabolizes resveratrol the more rapidly, levels of this stilbene being very low compared to control (18%) as soon as after 3 days of culture (Fig 6). Resveratrol was not metabolized in other known stilbenes, since resveratrol-derived compounds were not found in the fungi culture medium (data not shown). Compared to resveratrol, δ-viniferin was metabolized less rapidly. 30, 42 and 20% of added δ-viniferin were degraded after 6 days for *N*. *parvum* Bourgogne S-116, *N*. *parvum* Bt-67 and *D*. *seriata* 98.1 respectively. After 11 days, *N*. *parvum* Bourgogne S-116 was the fungi that degraded the less viniferin (52% remaining compared to control), followed by *N*. *parvum* Bt-67 (93.5% of degradation) and *D*. *seriata* (98.5% of degradation). It is interesting to note that inhibition of *N*. *parvum* Bourgogne S-116 growth (50% compared to control) by δ-viniferin is correlated with a lower δ-viniferin degrading activity. It is also likely that laccase and peroxidase activities that were identified in *D*. *seriata* and to a lesser extent in *N*. *parvum* participate in both resveratrol and δ-viniferin metabolization. The high laccase activity evidenced in *D*. *seriata* could explain the very low levels of both stilbenes after 11 days of growth (Fig 2A). In contrast, *N*. *parvum* Bt-67 is able to metabolize both resveratrol and δ-viniferin but is characterized by lower laccase and peroxidase activities compared to *D*. *seriata*, suggesting that this fungus has other enzymatic systems of phenolic metabolization.

Our results are consistent with other studies reporting that resveratrol was not very inhibitory to the growth of two different grapevine pathogens, *Plasmopara viticola* and *Botrytis cinerea* [75], whereas it inhibited the growth of *Phomopsis viticola*, but with a relatively low activity [42]. Pterostilbene and δ-viniferin were also previously described as the most effective on *P*. *viticola* [76–78]. Similarly, resveratrol had no inhibitory activity on *E*. *lata* growth, even at high concentration (4400 μM, [79]).

In a detailed study focusing on the *in vitro* antifungal activity of different phenolics towards trunk disease fungi, it was shown that *Botryosphaeriaceae* strains are generally sensitive to stilbenes at a 500 μM concentration [49]. However, among the different *Botryosphaeriaceae* strains, *N*. *parvum* was less affected than *D*. *seriata* that was more severely inhibited by stilbenoids, especially piceatannol, pterostilbene and ɛ-viniferin. The IC50 of ɛ-viniferin on *D*. *seriata* was evaluated at 250 μM [49]. Differences in fungi sensitivity towards stilbenes between our study and the study from Lambert et al. [49] could be explained by the use of a higher stilbene concentration (500 μM compared to 250 μM in our study) and the test of different *N*. *parvum* strains. Moreover, the activity of δ-viniferin was not evaluated in the above discussed work. In agreement with our experiments, stilbene activity was shown fungistatic and not fungicidal [49]. In another study [66], Djoukeng et al performed a disk diffusion antifungal assay and reported that stilbenoids (resveratrol, ε- and δ-viniferin) had no effect on *D*. *seriata*.

It should be pointed out that dimerization of resveratrol to δ-viniferin can be realized by fungi, such as *Botrytis cinerea* [52], and in the presence of purified laccases of *Trametes versicolor*, associated with white rot [80]. *Diplodia seriata* was also described as able to oxidize wood δ-resveratrol into the dimer δ-viniferin [66]. However, we were not able to detect any δ-viniferin in the medium of fungi supplemented with resveratrol. Furthermore, our results show that δ-viniferin seems to be degraded by *Botryosphaeriaceae* fungi.

To summarize, our study provides a better understanding of aggressiveness factors expressed by two major *Botryosphaeriaceae*. Each B. dieback associated fungi is characterized by a specific signature of secondary metabolites and secreted toxic protein production [22,27] and by differential wood decay enzymatic activity equipment (this work). The specific signature of *Botryosphaeriaceae* aggressiveness factors could explain the importance of fungi complexes in putative synergistic activity in order to fully colonize the host. *N*. *parvum* has a more efficient enzymatic activity for cellulose and hemicellulose degradation, whereas *D*. *seriata* has a more efficient enzymatic activity for lignin degradation. In addition, B. dieback associated fungi have the capacity to rapidly degrade stilbenes, and this capacity is likely important for the fungi aggressiveness. Indeed, *D*. *seriata* growth in the wood is slow, but higher laccase and peroxidase activities could enable this fungus to circumvent phytoalexin production and lignin barriers. Wood and phenolic compound decay enzymatic activities could explain that even if grapevine is able to perceive the invaders and to mount defense responses such as stilbene synthesis and cell wall reinforcement, B. dieback fungi are able to bypass these chemical and physical barriers. It would be interesting to further study the composition of cell wall polymers in susceptible *vs* tolerant trunk disease grapevine varieties.

## Supporting information

S1 Table Tables of data sets that were used for Figs 1–7. Table A: Data for polysaccharide degrading enzyme activities (Fig 1). Table B: Data for laccase and peroxidase activities (Fig 2). Table C: data of fungi growth in media containing viniferin (Fig 3). Table D: data of fungi growth in medium containing resveratrol (Fig 4). Table E: data of fungi growth in media containing δ-viniferin or resveratrol, one day before Petri dish saturation (Fig 5). Table F: data of resveratrol metabolization (Fig 6). Table G: data of δ-viniferin metabolization (Fig 7).
(XLSX)
